# Dynamic suction: a novel technique to optimize EUS-guided liver biopsy

**DOI:** 10.1016/j.vgie.2024.02.013

**Published:** 2024-04-07

**Authors:** David Cheung, Andy Lin, Khaldoon Khirfan, Usman Rahim, Amirali Tavangar, Frances Dang, Kenneth Chang, Jason Samarasena

**Affiliations:** 1 Division of Internal Medicine, University of California Irvine School of Medicine, Orange, California; 2 Division of Gastroenterology/Hepatology, University of California Irvine School of Medicine, Orange, California

## Background

Liver biopsy is an essential diagnostic procedure that offers prognostic information and informs treatment decisions for a variety of liver diseases. The adequacy of liver biopsy samples is the most important factor in interpreting liver pathology. The American Association for the Study of Liver Diseases guidelines defines an adequate specimen as having 11 complete portal tracts and a length of at least 1.5 cm.^1^ Failure to obtain an adequate specimen may necessitate the collection of additional specimens to further characterize the liver pathology.

Our group developed a novel technique for EUS-guided liver biopsy (EUS-LB) using “dynamic suction” (EUS-LB-D), which uses a variation of the wet heparin suction technique during tissue procurement.^2^^,^^3^ When executed properly, the technique consistently yields excellent and adequate liver biopsy samples.

## Technique

We provide comprehensive instruction for performing EUS-LB-D, as shown in Video 1 (available online at www.videogie.org). The first step is to prepare a 19-guage Franseen-tipped fine-needle biopsy (FNB) needle. The needle is primed with 3 mL of heparinized saline that is drawn up into a 20-mL stopcock syringe. Heparin is used to prevent the formation of blood clots within the sample that can fragment the sample. The needle is subsequently flushed with the heparinized saline until a few droplets exit the tip of the needle. The stopcock syringe is then closed, and 15 mL of suction is applied to the syringe. The stopcock syringe is subsequently removed from the needle, ready for use when suction is required in the technique. The FNB needle is then loaded into the working channel of the echoendoscope and placed into position for liver biopsy. During the first actuation, the needle is passed once with rapid throw without an attached stop-cock syringe or suction. The actuation without suction allows for the channel of heparin within the needle to be freely displaced, allowing for the liver specimen to be drawn up into the needle. Once the needle is fully extended to a premeasured length, the stopcock syringe with 15 mL of suction is reattached and suction is briefly applied for 2 seconds to “fix the needle.” This cuts the specimen from the liver parenchyma and brings the specimen even more proximally into the needle. Suction is kept on only briefly to prevent excessive blood from entering the needle. The needle is then withdrawn under eFlow Doppler to the reset position until it reaches just 1 to 2 cm below the liver capsule.

**Figure 1:**
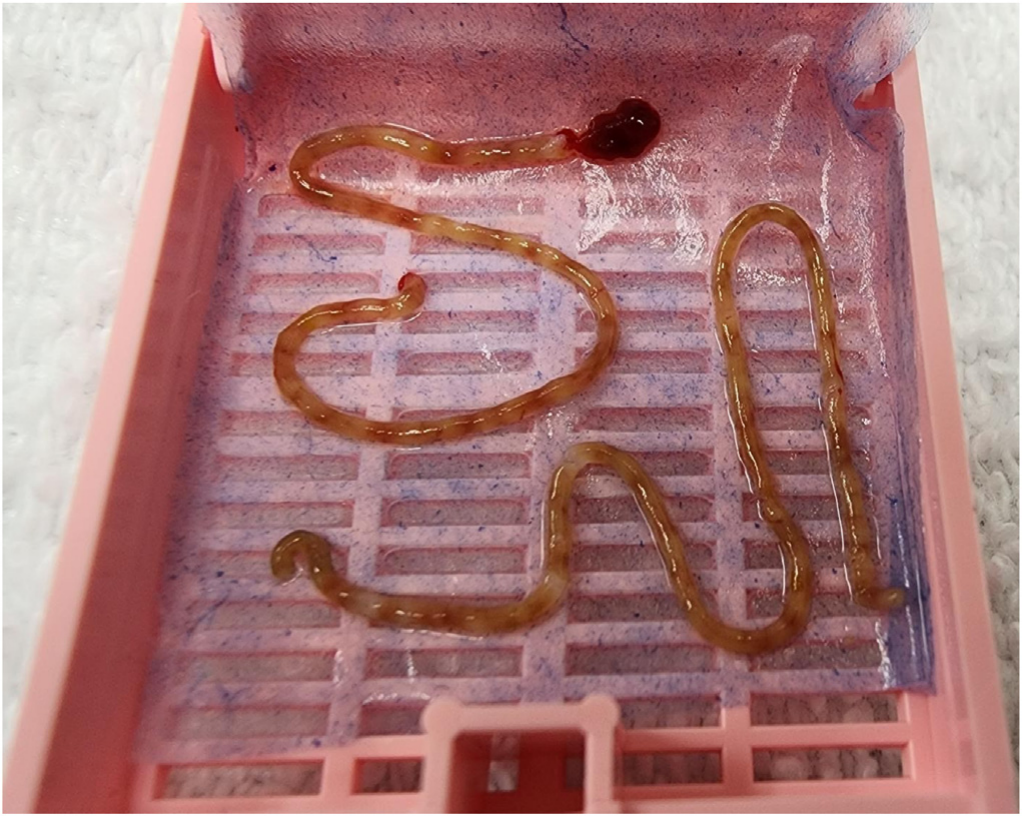
Gross specimens of liver biopsy. The longest core length of the samples was 5.9 cm, with a total core length of 16.1 cm. There was an average of 25.13 complete portal tracts within each sample.

**Figure 2:**
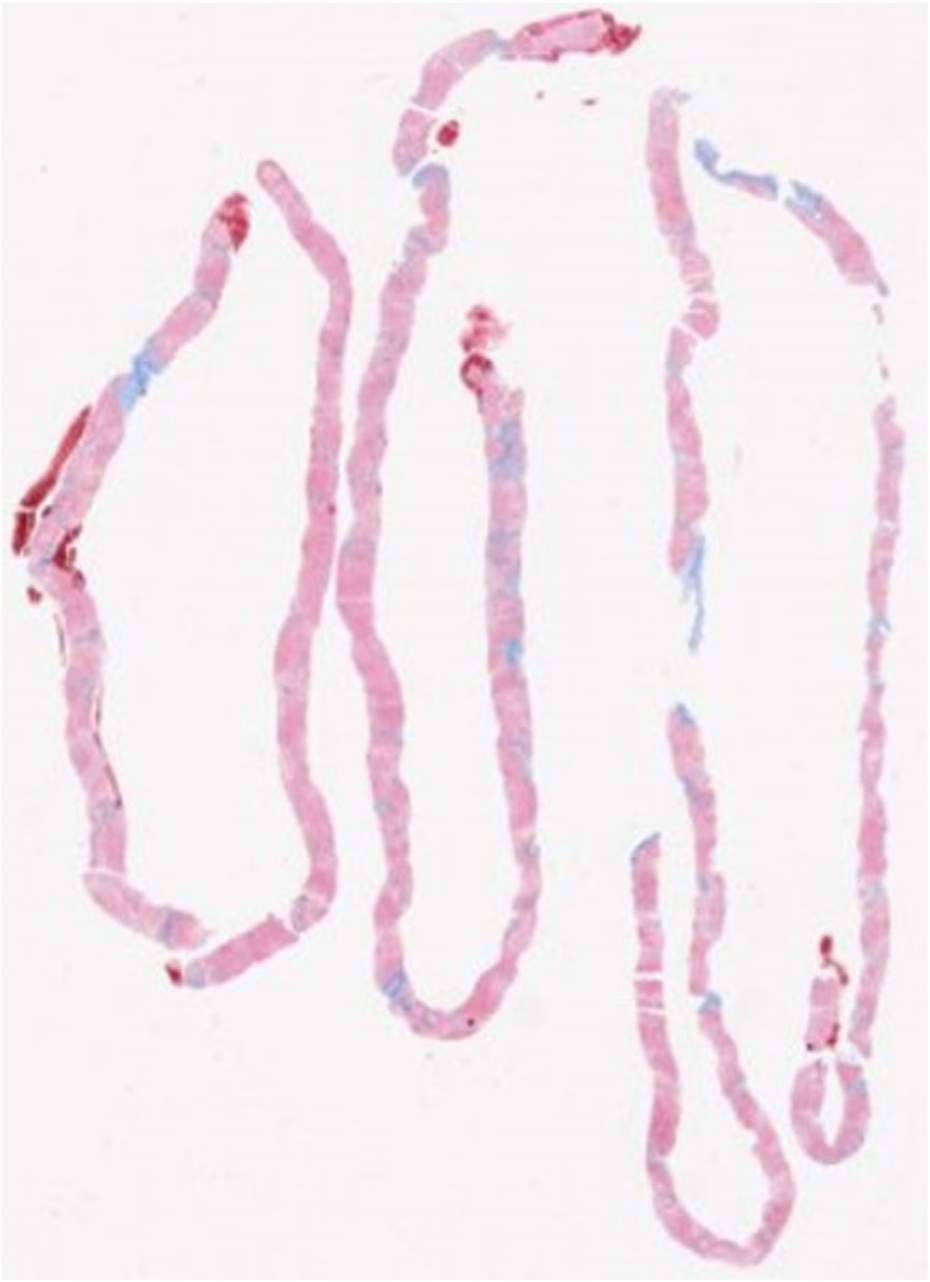
Histopathology specimen of EUS-guided liver biopsy.

During the second actuation, the suction is briefly reapplied at the beginning of the throw and is turned off once the needle is fully extended. These steps are repeated for the third actuation. A total of 3 actuations are performed within a single pass. Once complete, the FNB needle is withdrawn under color Doppler ultrasound for evaluation of bleeding. A 10-mL saline flush syringe is attached to the inlet of the 19-gauge needle to express the liver biopsy specimen out of the needle onto a plastic tray. Using a needle, we separate the blood clots from the specimen and place the specimen into a cassette that is lined with filter paper (Figs. 1 and 2).

## Results

The dynamic suction is a modification to the EUS-LB wet suction heparin technique that has yielded a dramatic improvement in liver-tissue acquisition at our center.^3^ From October 2021 to October 2022, a total of 30 patients underwent EUS-LB-D, resulting in a mean total specimen length of 6.45 cm and complete portal tract count of 25.13 per biopsy sample (Tables 1 and 2). These measurements far exceed the American Association for the Study of Liver Diseases criteria for liver biopsy adequacy and other published EUS-LB studies.^4^ Our single center had 100% technical and diagnostic success with no serious adverse events. All specimens were considered adequate for staging of fibrosis with 40% of samples demonstrating F4 fibrosis.

**Table 1:** Patient demographic data, liver biopsy indication, and METAVIR score

Factor (n = 30)	Percentage of patients
Demographic	
Male	56.7%
Female	43.3%
Indications	
NASH	56.7%
HBV	13.3%
HCV	10%
Alcohol-related liver disease	10%
Elevated LFT	3.3%
Other	6.7%
METAVIR score	
F0	26.6%
F1	13.3%
F2	13.3%
F3	6.7%
F4	40%

*HBV*, Hepatitis B virus; *HCV*, hepatitis C virus; *LFT*, liver function test; *NASH*, nonalcoholic steatohepatitis.

**Table 2:** Outcomes of the biopsy specimens obtained using EUS-guided liver biopsy with the dynamic suction technique

Outcome	Average ± SD (range)
Average fixed tissue length (cm)	6.45 ± 2.96 (2.6-16.1)
Longest single core length (cm)	5.9 ± 1.23 (1.3-5.9)
Average complete portal tract (n)	25.13 ± 11.32 (8-50)
Technical success	30/30 (100%)
Diagnostic adequacy	30/30 (100%)
Serious adverse events	0/30 (0%)

## Conclusion

The dynamic suction technique is a simple modification to the traditional wet heparin suction technique and achieves exceedingly adequate specimens. We believe that the dynamic suction technique is an effective and reliable technique for adequate tissue acquisition for EUS-guided liver biopsy.

## Disclosure

Dr Chang is a consultant for Olympus, Cook Medical, Endogastric Solutions, and Medtronic. Dr Samarasena is a consultant for Medtronic, Olympus, CONMED, Applied Medical, Neptune Medical, and Boston Scientific, and is a shareholder of Satisfai Health. All other authors disclosed no financial relationships relevant to this publication.

## Supplementary data

Video 1 Description and demonstration of EUS-guided liver biopsy using the dynamic suction technique.
